# A Cross-Sectional Study Comparing Patient Education Guides Created by ChatGPT and Google Gemini for Common Cardiovascular-Related Conditions

**DOI:** 10.7759/cureus.77442

**Published:** 2025-01-14

**Authors:** Nayanaa Varsaale, Collin R George, Lakshmi Manasa Gunturi, Hariharasudhan Saravanan, Saswaath Thiruvengadam K, Gayatri Anilkumar Menon

**Affiliations:** 1 General Surgery, Capital Medical University, Beijing, CHN; 2 Emergency Medicine, Jubilee Mission Medical College and Research Institute, Thrissur, IND; 3 General Surgery, Government General Hospital, Rangaraya Medical College, Kakinada, IND; 4 Internal Medicine, Government Stanley Medical College and Hospital, Chennai, IND; 5 Internal Medicine, Sri Ramachandra Medical College and Research Institute, Chennai, IND; 6 Internal Medicine, Azeezia Institute of Medical Sciences and Research, Kollam, IND

**Keywords:** artificial intelligence, chatgpt, google gemini, hypertension, hypertriglyceridemia, metabolic syndrome, obesity, patient education brochure

## Abstract

Introduction

Obesity, hypertension, and hypertriglyceridemia are key components of metabolic syndrome, a major contributor to cardiovascular diseases (CVDs), which remain a leading cause of global mortality. Patient education on these conditions can empower individuals to adopt preventive measures and manage risks effectively. This study compares ChatGPT and Google Gemini, two prominent artificial intelligence (AI) tools, to evaluate their utility in creating patient education guides. ChatGPT is known for its conversational depth, while Google Gemini emphasizes advanced natural language processing. By analyzing readability, reliability, and content characteristics, the study highlights how these AI tools cater to diverse patient needs, aiming to enhance health literacy outcomes.

Methodology

A cross-sectional study evaluated patient education guides on obesity, hypertension, and hypertriglyceridemia, focusing on their links to metabolic syndrome. Responses from ChatGPT and Google Gemini were analyzed for word count, sentence count, readability (using the Flesch-Kincaid calculator), similarity score (using Quillbot), and reliability score (using the modified DISCERN score), with statistical analyses performed using the R Version 4.3.2.

Results

Statistical analysis revealed a significant difference in word and sentence counts between the AI tools: ChatGPT averaged 591.50 words and 66 sentences, while Google Gemini had 351.50 words and 36 sentences (p = 0.001 and p < 0.0001). However, the average words per sentence, average syllables per word, grade level, similarity percentage, and reliability scores did not differ significantly. Although Google Gemini had a higher ease score (41.75) compared to ChatGPT (34.10), this difference was not statistically significant (p = 0.080). Both tools exhibited similar readability and reliability, indicating their effectiveness for patient education, despite ChatGPT providing longer responses.

Conclusion

The study found no significant difference between the two AI tools in terms of ease, grade, and reliability scores, with no correlation between ease and reliability scores.

## Introduction

Cardiovascular disease (CVD) is the leading cause of morbidity and mortality globally. One major contributor to the development of CVD is metabolic syndrome [1]. Obesity, hypertension, and hypertriglyceridemia are key components of metabolic syndrome. Metabolic syndrome is a combination of metabolic irregularities that include centrally distributed obesity, decreased high-density lipoprotein cholesterol (HDL-C), elevated triglycerides, elevated blood pressure (BP), and hyperglycemia [1]. Every component of MetS is an independent risk factor for the development of CVD and an aggregate of these risk factors leads to a propionate severity of CVD, related to a spectrum of conditions including microvascular dysfunction, coronary atherosclerosis, myocardial infarction, and heart failure [2]. Patient education can be used to promote awareness of the disease which may help in the early identification of risk factors and complications and improve compliance with medications and increase lifestyle modifications which may reduce the overall morbidity and mortality of the disease. 

ChatGPT (OpenAI, San Francisco, USA, 2022) is an artificial intelligence (AI) language model known for its humanlike conversational abilities [3]. Gemini Open (Google Deepmind, CA) was introduced in 2023 with advanced natural language processing (NLP) and can provide healthcare information to patients. These chatbots now offer a viable alternative to traditional search engines like Google for health education for patients. They offer round-the-clock accessibility and comprehensive information in a single platform, thus improving health literacy [4]. While patient education guides provided by these sophisticated language bots are more comprehensible, they were found to be less readable depending on individual health literacy when compared to traditional search engines [5]. 

With the increasing reliability of AI for patient education for various diseases to improve health literacy, this study was undertaken to compare responses generated by ChatGPT and Google Gemini in creating patient education guides on common conditions such as obesity, hypertension, and hypertriglyceridemia and highlight their connections to metabolic syndrome. 

Aims and objectives

This study aims to compare the responses generated by ChatGPT and Google Gemini in creating patient education guides on common conditions such as obesity, hypertension, and hypertriglyceridemia, highlighting their connections to metabolic syndrome, based on readability and ease of understanding. 

## Materials and methods

A cross-sectional original research study was conducted over one week from September 2, 2024, to September 9, 2024. Since no human participants were involved in this study, Ethics Committee Approval was not required. 

The data was collected in the following manner: Three conditions were selected such as obesity, hypertension, and hypertriglyceridemia, focusing on their links to metabolic syndrome in the internal medicine specialty. Two AI tools were selected, namely, ChatGPT (Version 3.5) and Google Gemini (Version 1.5 Flash) on September 3, 2024, for the generation of brochures for patient education. Prompts were given to both AI tools: Write a patient education guide for abdominal obesity and its role in metabolic syndrome. Responses from ChatGPT and Google Gemini were analyzed and collected in a Microsoft Word document. The Flesch-Kincaid calculator was used for word count, sentence count, ease of understanding, and grade level of the information generated [6]. QuillBot Plagiarism Tool was used for analyzing similarity [7].

Reliability of scientific text was analyzed using a modified DISCERN score which was 2 for both ChatGPT and Google Gemini. A modified DISCERN score was designed to assess the quality and reliability of health information. This modified score typically involves adjustments to the original content types, while still evaluating key aspects like clarity, accuracy, and relevance. Statistical analysis was performed using R Version 4.3.2 [8]. 

Lastly, statistical analysis was undertaken and data was exported to MS Excel (Microsoft Corporation, Redmond, Washington, United States). Statistical analysis was done using R Version 4.3.2. R is a language and environment for statistical computing (R Foundation for Statistical Computing, Vienna, Austria). 

The responses generated by ChatGPT and Google Gemini were compared using an unpaired T-test, and a p-value of <0.05 was considered significant. The correlation between ease score and reliability score was compared using Pearson’s correlation coefficient.

## Results

In this cross-sectional study, ChatGPT and Google Gemini were utilized to develop patient information guides on common cardiovascular-related conditions like metabolic syndrome, abdominal obesity, insulin resistance, hypertension, elevated triglycerides, and high blood glucose levels. To assist patients in managing their conditions through lifestyle modifications and medical interventions, the AI-generated guides provided clear, understandable information that simplified complicated medical concepts. The educational materials generated by both platforms were precise and focused on the patient. This study demonstrates how AI-powered tools can be used to create excellent patient education materials that improve patients' comprehension and control of cardiovascular risks and associated conditions. 

An independent sample T-test has been used to compare the means between ChatGPT and Google Gemini. The normality of the variables was assessed using the Shapiro-Wilk Test, and the equality of variances was tested using Levene’s test. Based on the p-values obtained in Table 1, there is a statistically significant difference between words and sentences generated by the two AI tools.

**Table 1:** Characteristics of responses generated by ChatGPT and Google Gemini

	ChatGPT		Google Gemini		p-value^+ ^
	Mean	Standard deviation	Mean	Standard deviation	
Words	591.50	85.16	351.50	28.12	0.001*
Sentences	66.00	6.51	36.00	4.00	<0.000*
Average words per sentence	9.05	1.80	9.83	0.92	0.365
Average syllables per word	1.93	0.10	1.83	0.05	0.060
Grade level	10.75	1.27	9.88	0.68	0.173
Ease score	34.10	8.53	41.75	4.44	0.080
Similarity %	42.00	15.66	51.88	22.55	0.399
Reliability score	3.00	0.89	2.50	0.55	0.270
^+^T-test. P-values < 0.05 are considered statistically significant					

The responses produced by ChatGPT and Google Gemini are shown in Table 1, which also emphasizes variations in multiple metrics. Statistical analysis found between the two AI tools showed significant differences between word and sentence counts; ChatGPT produced an average of 591.50 words and 66 sentences, while Google Gemini produced an average of 351.50 words and 36 sentences (p = 0.001 and p < 0.0001, respectively). The average number of words per sentence, however, did not differ significantly (p = 0.365), suggesting that the two platforms' sentence structures were similar. The analysis also evaluated additional characteristics, including grade level, ease score, similarity percentage, reliability score, and average syllables per word. Despite having a higher ease score (41.75) than ChatGPT (34.10), Google Gemini and ChatGPT did not differ statistically significantly (p = 0.080). Average syllables per word (p = 0.060), grade level (p = 0.173), similarity percentage (p = 0.399), and reliability score (p = 0.270) did not differ significantly. 

**Figure 1:**
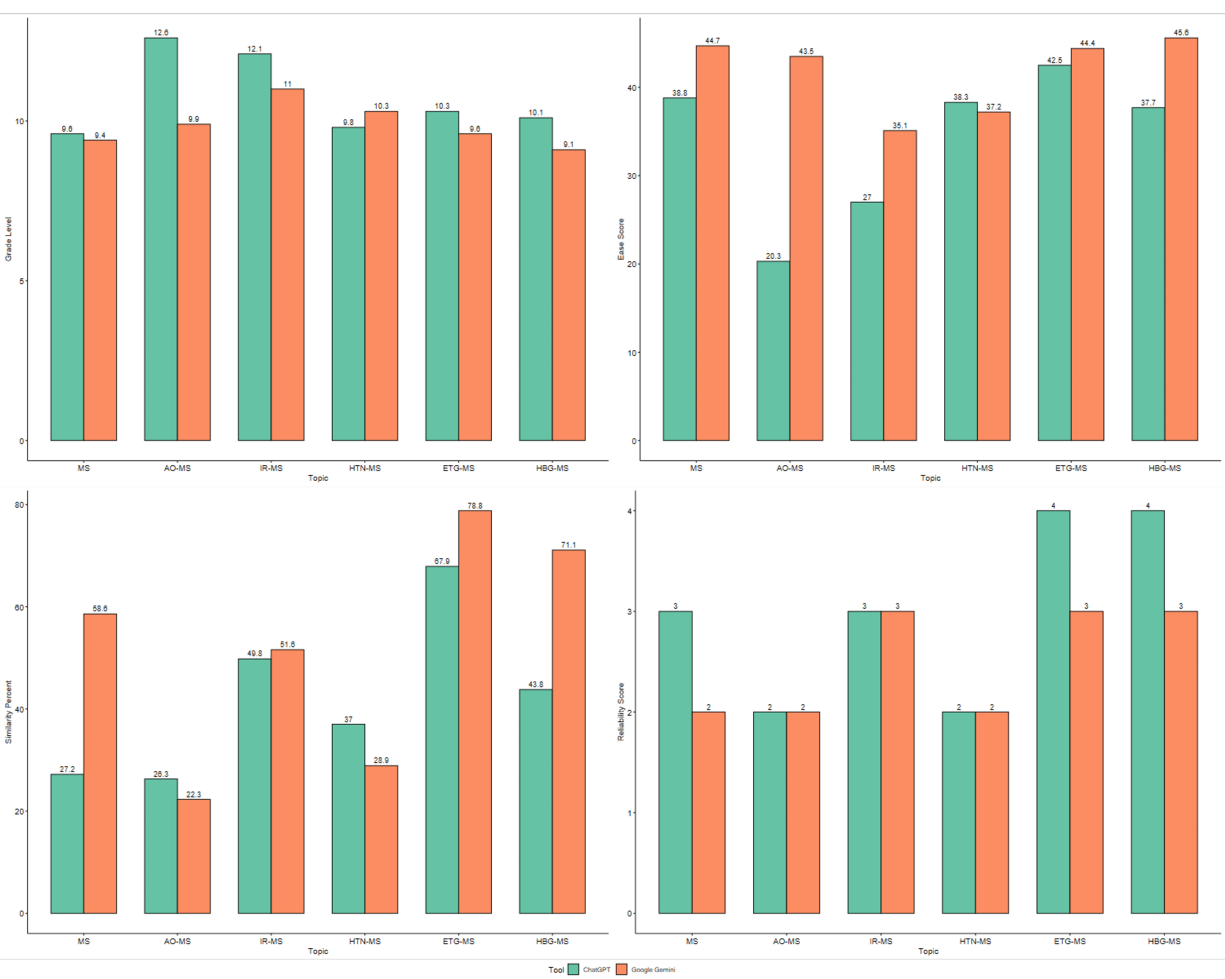
Graphical representation of comparison between grade level, ease score, similarity percent, and reliability score, for the patient education guide generated by ChatGPT and Google Gemini MS: metabolic syndrome; AO-MS: abdominal obesity and its role in metabolic syndrome; IR-MS: insulin resistance and its role in metabolic syndrome; HTN-MS: hypertension and its role in metabolic syndrome; ETG-MS: elevated triglycerides and its role in metabolic syndrome; HBG-MS: high blood glucose levels and its role in metabolic syndrome

As shown in Figure 1, the comparison between ChatGPT patient education guides and Google Gemini revealed significant differences in four key metrics: grade level, ease score, similarity percent, and reliability score. Google Gemini consistently produced guides with higher grade levels, indicating a more complex writing style, whereas ChatGPT maintained lower grade levels, allowing for better readability and broader accessibility. In terms of ease scores, Google Gemini outperformed on most topics, implying that its content was easier for patients to understand. Furthermore, Google Gemini showed significantly higher similarity percentages, particularly for topics such as elevated triglycerides (ETG-MS) and high blood glucose (HBG-MS), indicating a better fit with validated educational materials. Both tools, however, achieved comparable reliability scores of 3 to 4 across all topics, indicating high factual accuracy. These findings indicate that while Google Gemini may be more effective at producing standardized and patient-friendly materials, ChatGPT excels at creating accessible content for diverse populations with varying literacy levels. 

In summary, the findings show that both tools produce similar readability and reliability, indicating their efficacy as patient education resources, even though ChatGPT produces longer responses. Further investigation could explore the implications of these findings on user comprehension and engagement.

## Discussion

Metabolic syndrome remains a critical and escalating public health issue, with over 15,000 articles published since May 2001, with an average of more than 40 articles each week [9]. Patient education has shown remarkable potential in improving the individual components of metabolic syndrome, often surpassing the effectiveness of traditional management approaches [10].

AI-powered chatbots rely on natural language processing to interpret user input, identify the underlying intent, and provide contextually appropriate responses. By simulating humanlike conversation, these tools aim to deliver information in a comprehensible and engaging manner. In healthcare, AI chatbots have the potential to revolutionize patient education by providing accurate, personalized information instantly, thereby bridging the gap between complex medical knowledge and patient understanding. These systems can tailor explanations to individual needs, adapt to different literacy levels, and offer multilingual support to reach diverse populations [11].

The effectiveness of these chatbots can be further enhanced through various techniques, including expanding their training datasets to improve accuracy, customizing the system for specific domains such as healthcare, and refining algorithms through user feedback. Additionally, integrating methods like active learning and incorporating human oversight ensures that chatbots remain relevant and accurate, even in complex or nuanced scenarios. By providing round-the-clock accessibility and reducing the burden on healthcare providers, AI chatbots can play a pivotal role in empowering patients to make informed decisions about their health.

The present paper is a cross-sectional study comparing the number of words and sentences produced by two AI tools, ChatGPT and Google Gemini, for patient education guides on metabolic syndrome and the role of elevated triglycerides, insulin resistance, hypertension, abdominal obesity, and high blood glucose levels in metabolic syndrome, revealing a statistically significant difference. AI provides solutions in the form of virtual consultations, language translation tools, and customized educational materials. By ensuring information consistency across practices, AI enhances patient comprehension and the overall experience [12]. The ability to provide individualized health information is one of the main advantages of AI in healthcare. AI algorithms can analyze patient data, including lifestyle factors and medical histories, to give patients personalized advice on how to stay healthy. Patients can make more informed decisions about their care and gain a better understanding of their health with the use of this information [13]. 

For patients to read, understand, and retain the information given in the guide better, it needs to be short and concise. This study uses parameters including the average number of words and sentences, average word count per sentence, average syllables per word, and Flesch Reading Ease score. The Flesch Reading Ease gives a text a score between 1 and 100, with 100 being the highest readability score. In this study, the mean Flesch Reading Ease score was 34.10 and 41.75 in ChatGPT and Google Gemini AI tools, respectively. This indicates that the text is difficult to read and is best understood by college graduates. The National Institutes of Health (NIH) and the National Academy of Medicine (NAM) have advised that patient education materials be crafted at or below a sixth-grade reading level [14]. Due to their reliance on pre-existing literature for training, AI language models like ChatGPT and Google Gemini may produce content with similar phrasing, raising concerns about unintentional plagiarism. In medical science, plagiarism is particularly detrimental, as it compromises the originality and credibility of educational resources and risks diminishing the trust of patients and healthcare providers. This study found an average similarity of 42.00% (SD = 15.66) for ChatGPT-generated content and 51.88% (SD = 22.55) for Google Gemini, with a T-test yielding a p-value of 0.399. This indicates no statistically significant difference between the two tools in terms of similarity levels. These findings align with similar studies on AI-generated medical content, highlighting the need for rigorous originality checks to ensure AI-produced patient education materials maintain ethical standards and credibility. 

The modified DISCERN score is a tailored version of the DISCERN questionnaire, specifically adapted to evaluate the reliability and quality of health information available online or through digital media. It provides a standardized approach to assess how well information meets the needs of consumers and adheres to evidence-based standards [15]. In this study, the reliability scores of contents generated by AI tools were compared using the modified DISCERN score. ChatGPT achieved a mean score of 3.00 (SD = 0.89), while Google Gemini scored slightly lower with a mean of 2.50 (SD = 0.55). The T-test yielded a p-value of 0.270, indicating no statistically significant difference between the two tools in terms of reliability (p-values of < 0.05 are considered significant). These findings are consistent with other studies exploring the reliability of AI-generated health information, emphasizing the need for further refinement in AI tools to enhance the quality and trustworthiness of their outputs. 

Limitations

While this study provides valuable insights, several limitations should be considered. First, the analysis was restricted to only two AI tools-ChatGPT 3.5 and Google Gemini, leaving the performance of other AI platforms unexplored. Expanding the scope to include additional tools would provide a more comprehensive understanding of AI-generated health education materials. Second, the study focused on a limited number of diseases, assessing only six conditions related to metabolic syndrome. Including a broader range of medical topics could yield more generalizable insights. Additionally, the version of ChatGPT used in this study, version 3.5, is not the latest iteration and may lack updated content or improved functionality available in newer versions. This raises concerns about how well AI tools keep pace with advances in medical science and their ability to deliver the most current and accurate information to users. Future studies should address these gaps by examining multiple AI tools, covering a wider array of medical conditions and using updated versions of the models to ensure relevance and reliability.

## Conclusions

This study identified no significant difference in the average ease score, grade score, and reliability scores of responses generated by the two AI tools for patient information brochures on obesity, hypertension, and hypertriglyceridemia. There is no correlation between ease and reliability scores for the two software. Further studies are needed to explore the use of AI techniques in many diseases, including current ones and for medications. It is important to evaluate whether these technologies follow the most recent criteria when creating content to improve tools to give up-to-date reliable information. Once suitable, the authors suggest these tools should be freely available to the population at large. 
